# Testing and Validation of High Density Resequencing Microarray for Broad Range Biothreat Agents Detection

**DOI:** 10.1371/journal.pone.0006569

**Published:** 2009-08-11

**Authors:** Tomasz A. Leski, Baochuan Lin, Anthony P. Malanoski, Zheng Wang, Nina C. Long, Carolyn E. Meador, Brian Barrows, Sofi Ibrahim, Justin P. Hardick, Mohamed Aitichou, Joel M. Schnur, Clark Tibbetts, David A. Stenger

**Affiliations:** 1 Center for Bio/Molecular Science and Engineering, Code 6900, Naval Research Laboratory, Washington D. C., United States of America; 2 Nova Research Incorporated, Alexandria, Virginia, United States of America; 3 National Research Council- Naval Research Laboratory (NRC-NRL), Washington D. C., United States of America; 4 The United States Army Medical Research Institute of Infectious Diseases (USAMRIID), Ft. Detrick, Frederick, Maryland, United States of America; 5 Akimeka, Frederick, Maryland, United States of America; 6 College of Science, George Mason University, Fairfax, Virginia, United States of America; 7 TessArae, LLC, Potomac Falls, Virginia, United States of America; U.S. Naval Medical Research Center Detachment/Centers for Disease Control, United States of America

## Abstract

Rapid and effective detection and identification of emerging microbiological threats and potential biowarfare agents is very challenging when using traditional culture-based methods. Contemporary molecular techniques, relying upon reverse transcription and/or polymerase chain reaction (RT-PCR/PCR) provide a rapid and effective alternative, however, such assays are generally designed and optimized to detect only a limited number of targets, and seldom are capable of differentiation among variants of detected targets. To meet these challenges, we have designed a broad-range resequencing pathogen microarray (RPM) for detection of tropical and emerging infectious agents (TEI) including biothreat agents: RPM-TEI v 1.0 (RPM-TEI). The scope of the RPM-TEI assay enables detection and differential identification of 84 types of pathogens and 13 toxin genes, including most of the class A, B and C select agents as defined by the Centers for Disease Control and Prevention (CDC, Atlanta, GA). Due to the high risks associated with handling these particular target pathogens, the sensitivity validation of the RPM-TEI has been performed using an innovative approach, in which synthetic DNA fragments are used as templates for testing the assay's limit of detection (LOD). Assay specificity and sensitivity was subsequently confirmed by testing with full-length genomic nucleic acids of selected agents. The LOD for a majority of the agents detected by RPM-TEI was determined to be at least 10^4^ copies per test. Our results also show that the RPM-TEI assay not only detects and identifies agents, but is also able to differentiate near neighbors of the same agent types, such as closely related strains of filoviruses of the Ebola Zaire group, or the Machupo and Lassa arenaviruses. Furthermore, each RPM-TEI assay results in specimen-specific agent gene sequence information that can be used to assess pathogenicity, mutations, and virulence markers, results that are not generally available from multiplexed RT-PCR/PCR-based detection assays.

## Introduction

Deliberate release of a virulent biological agent in a densely populated area can have devastating effects. Early detection of an attack that uses biowarfare agents is extremely difficult, in part because diagnosis may be confounded by nonspecific “flu-like” initial symptoms [1], [2], coupled with very small *a priori* likelihood of such exposures and etiologies of infection. Rapid and effective methods for accurate and sensitive detection of biothreat agents are critical elements for national security. Traditional methods of identification of infectious agents based on culture, although reliable and familiar, are too slow to be relevant in the case of an intentional release of a biological agent. Additionally the safety considerations limit culture-based assays for those agents to a few facilities that are able to assure safety and containment of such agents. The fact that a significant proportion of microorganisms are not amenable to culture [3] is another serious drawback of those techniques. Finally, one of the most significant challenges to the successful detection of biowarfare agents is their diversity. Potential biothreat agents can be found across a number of bacterial and viral taxonomic groups [4]. Furthermore, many biothreat agents are very similar to relatively harmless species [5], [6]. An ability to distinguish innocuous genetic near-neighbors from biothreat agents would lower the false alarm rate, which is crucial in risk management, and successful public health response.

Molecular methods such as RT-PCR/PCR may provide rapid identification based on the direct detection of bacteria and viruses in clinical or environmental samples, and thus address the issues of speed of assay. However, most current detection technologies in use are optimized for the detection of a single or a limited number of pathogens. In general such assays rely upon short nucleic acid sequence signature elements to detect and identify the specific targets of each assay. This rationale imposes a contradictory challenge to optimize assay specificity (minimize false positive results) and sensitivity (minimize false negative results).

There are a number of attempts under way to develop technologies for broad-spectrum detection of infectious agents for clinical as well as biodefense applications [7], [8], [9], [10], [11], [12], [13], [14], [15]. One promising technology is the resequencing pathogen microarray (RPM). A number of recent studies using RPM technology have shown that it allows simultaneous detection of a large number of targeted infectious agents, retaining high specificity and clinically relevant sensitivity at a relatively modest cost [16], [17], [18], [19]. In addition, the architecture of resequencing microarrays allows for detection and identification of natural or engineered sequence variations of targeted agents. Sequences differing up to 15 percent from the prototype sequence on the chip can be reliably detected [20] and the resolution of individual bases allows for strain discrimination and detection of novel sequence variants [21]. A prototype resequencing pathogen microarray version 1 (RPM v.1) was designed and studied in our laboratory, primarily for detection of common respiratory pathogens plus six CDC category A biothreat agents [17]. It was demonstrated that RPM v.1 was able to identify intended targets and differentiate them from near neighbor species [22]. Building upon this experience, this paper describes the results for a new microarray design that covers a much broader range of potential biowarfare agents. This new microarray contains targets intended for detection of the majority of CDC category A, B and C select agents and a number of toxin genes.

While designing the multiple-pathogen microarray and its amplification protocol are critical tasks, collecting material for validating the multi-pathogen microarray is just as important and challenging. This is particularly true in the case of a microarray intended to detect biothreat agents, since the majority of its targets are not only classified as “select agents” but also potentially lethal. Such agents require handling in facilities with biosafety level ratings BSL-3 or BSL-4. For some agents such as *Bacillus anthracis* or *Yersinia pestis*, it is possible to obtain nucleic acids, avirulent strains or inactive organisms. However for the majority of agents required to validate the microarray, access even to their genomic nucleic acids is limited to specialized high security laboratories. To overcome this limitation, we developed an innovative validation strategy, which takes advantage of synthetic gene templates to establish the limit of detection (LOD), for every target on the microarray for which genomic templates are not available. Although synthetic DNA is routinely used in many areas of biomedical research and examples of application of synthetic templates for diagnostic assay validation [12], [23], [24] as well as attempts to create multivalent synthetic test templates [25], [26] can be found in scientific literature, this is the first report of a large scale validation strategy based primarily on synthetic genes. The results of this study show that by applying this strategy it is possible to develop and fine-tune the amplification protocol of the microarray to achieve target LOD. This validation is not a complete clinical validation which is expensive and difficult to implement but a “sensitivity” validation which ensures that the developed microarray and protocol is likely to perform well for clinical use. Retrospective testing of some targets using genomic templates demonstrated concordant results to those observed using synthetic templates. This study demonstrates that synthetic templates are suitable alternatives for the validation of multiple-pathogen microarrays and establishing LOD.

## Materials and Methods

### RPM-TEI chip design

The RPM-TEI arrays (TessArray^®^ RPM-TEI 1.0, TessArae LLC, Potomac Falls, VA) were designed to maximize detection coverage of CDC category A, B, and C biothreat agents. A total of 187 diagnostic sequences from 84 pathogens (including their subtypes) were selected and used to create RPM-TEI, which allows resequencing of 117 kb (see supporting materials: Table S1 and Figure S1).

**Figure 1:**
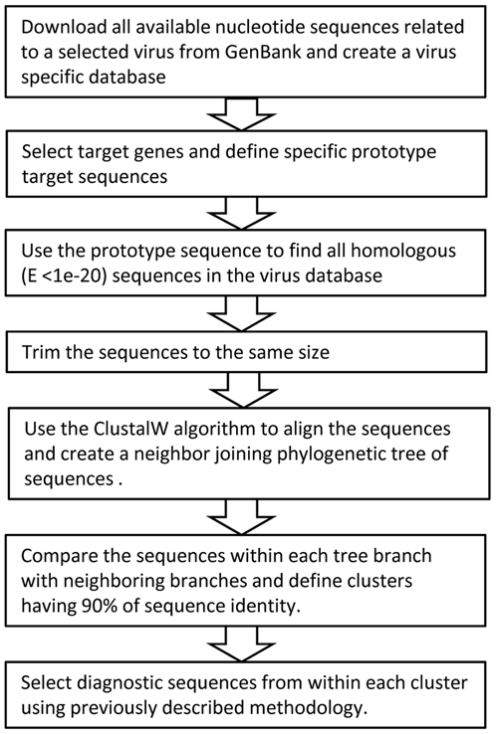
Selection of detector sequences used in RPM-TEI design. The diagram illustrates the main stages of the detector sequence selection process.

The design and target selection strategy used is described in detail in previous studies [20], [27] but has incorporated an expanded pre-processing step for highly variable organisms (Figure 1). The purpose of this step is to simplify the process of diagnostic sequence selection by defining subgroups of related sequences within a large set of sequences for particular target. Then each of those smaller groups is analyzed separately to find the minimum number of probes necessary to detect all of the sequences using a previously developed methodology.

The pre-processing is done by analyzing all the available sequences for a particular target in an organism that are available in GenBank. The sequences are downloaded, trimmed to the same length, and used to construct a phylogenetic tree. Finally the sequences on closely related branches of the tree are compared to define clusters with >90% of sequence identity. A single prototype detector sequence is initially selected from each cluster with the assumption that it will be able to detect all of the sequences in the cluster, since previous studies have demonstrated that RPM assays can reliably detect target sequences with as much as 15% variation [20].

The final step employs previously described algorithms [20], [27] to verify complete coverage of the cluster and select additional probes if required to ensure full coverage. For a detailed example, the procedure used to select probes for Lassa viruses is described in supporting methods Text S1.

### Primer selection

To simplify primer design and multiplex PCR optimization, four independent multiplex primer cocktails were developed for amplification of 187 targeted sequences represented on RPM-TEI array. The gene-specific primer pairs for all targets on the RPM-TEI chips (supporting Table S1) were designed according to the criteria described previously [18], [19]. Of the four multiplex primer mixes, mix I was dedicated primarily to bacterial targets and a few DNA viruses. Two primer mixes were designed to amplify mostly hemorrhagic fever viruses; mix II for those mainly endemic in Africa and Australia and III for those endemic in the Americas and additionally included Crimean-Congo hemorrhagic fever virus. The mix IV provides amplification of confounders and other agents. For list of agents included in each PCR group, see supporting Table S2.

### Multiplex RT-PCR Amplification

The multiplex reverse transcription (RT)-PCR amplification reactions were performed under conditions that have been previously described [18], [20]. The RT reaction products were subdivided for four different multiplex PCR amplification reactions. The amplified products from all four PCR reactions were combined again into a single sample and subjected to purification and processing prior to hybridizing to the RPM-TEI chips.

### Strains and templates

Control reference strains and field strains used to test the sensitivity and specificity of RPM-TEI and their sources are listed in Table 1. Since most of the agents that RPM-TEI was designed to detect required BSL-3 or BSL-4 facilities for safe handling, they were substituted for analytic sensitivity testing by synthetic DNA fragments manufactured by BlueHeron Biotechnology, Inc., Bothell, WA (see supporting Table S3 for a complete list). Viral strains tested with RPM-TEI at the United States Army Medical Research Institute for Infectious Diseases (USAMRIID, Frederick, MD) are listed in Table 2.

**Table 1:** Pathogens used as a source of nucleic acids for microarray validation.

Pathogen	Strain	Form	Source^1^
*Cryptosporidium parvum*	TU502	Nucleic acid	NRL
*Bacillus anthracis*	Ames	Nucleic acid	AFIP
*Bacillus cereus*	ATCC 14579	Live cells	ATCC
*Bartonella quintana*	ATCC 51694	Live cells	ATCC
*Campylobacter jejuni*	ATCC 700819D-5	Live cells	ATCC
*Clostridium perfringens*	ATCC 13124	Live cells	ATCC
*Clostridium tetani*	ATCC 9441	Live cells	ATCC
*Escherichia coli* O157:H7	ATCC 43985 (CDC EDL933)	Nucleic acid	ATCC
*Francisella tularensis*	SHU4	Nucleic acid	AFIP
*Leptospira interrogans*	ATCC 23478	Live cells	ATCC
*Mycobacterium tuberculosis*	ATCC 25177	Attenuated cells	ATCC
*Salmonella enterica*	ATCC 19430	Live cells	ATCC
*Vibrio cholerae*	ATCC 513940	Nucleic acid	ATCC
*Yersinia pestis*	D27	Nucleic acid	AFIP
Dengue type 2	ATCC VR-345	Live virus	ATCC
Dengue type 3	ATCC VR-1256	Live virus	ATCC
Dengue type 4	ATCC VR-1257	Live virus	ATCC
Human herpesvirus 1	ATCC VR-1493	Live virus	ATCC
Human herpesvirus 2	ATCC VR-734	Live virus	ATCC
Influenza A virus (H5N1)	CDC influenza A/H5N1	Live virus	CDC

1 NRL = Naval Research Laboratory, Washington, DC; ATCC = American Type Culture Collection, Manassas, VA; AFIP = Armed Forces Institute of Pathology, Washington, DC.; CDC = Centers for Disease Control, Atlanta, GA.

**Table 2:** Results of testing of the RPM-TEI using genomic preparations of selected viral agents.

Pathogen	Taxon	PCR group	Concentration	Identification result
Ebola Zaïre	Filoviridae	II	1 ng	Zaïre Ebola virus strain Zaïre 1995
Ebola Zaïre	Filoviridae	II	10^−1^ ng	Zaïre Ebola virus strain Zaïre 1995
Ebola Zaïre	Filoviridae	II	10^−2^ ng	Zaïre Ebola virus strain Zaïre 1995
Ebola Zaïre	Filoviridae	II	10^−3^ ng	Zaïre Ebola virus strain Zaïre 1995
Ebola Zaïre	Filoviridae	II	10^−4^ ng	Zaïre Ebola virus strain Zaïre 1995
Ebola Zaïre	Filoviridae	II	10^−5^ ng	No detection
Ebola Reston	Filoviridae	II	1 ng	Reston Ebola virus strain Pennsylvania
Ebola Reston	Filoviridae	II	10^−1^ ng	Reston Ebola virus strain Pennsylvania
Ebola Reston	Filoviridae	II	10^−2^ ng	Reston Ebola virus strain Pennsylvania
Ebola Reston	Filoviridae	II	10^−3^ ng	Reston Ebola virus strain Pennsylvania
Ebola Ivory Coast	Filoviridae	II	10^−1^ ng	Cotê d'Ivoire Ebola virus
Ebola Zaïre strain Mayinga	Filoviridae	II	10^−1^ ng	Zaïre Ebola virus strain Mayinga
Marburg Ravn	Filoviridae	II	10^−1^ ng	Lake Victoria Marburg virus strain Ravn
Marburg Musoke	Filoviridae	II	10^−1^ ng	No detection
Marburg Ci67	Filoviridae	II	10^−1^ ng	Lake Victoria Marburg virus strain Ci67
Lassa Josiah	Arenaviridae	II	1 ng	Lassa virus strain Josiah
Lassa Josiah	Arenaviridae	II	10^−1^ ng	Lassa virus strain Josiah
Lassa Josiah	Arenaviridae	II	10^−2^ ng	Lassa virus strain Josiah
Lassa Josiah	Arenaviridae	II	10^−3^ ng	No detection
Lassa Z148	Arenaviridae	II	1 ng	Lassa virus strain Z148
Lassa Z148	Arenaviridae	II	10^−1^ ng	Lassa virus strain Z148
Lassa Z148	Arenaviridae	II	10^−2^ ng	Lassa virus strain Z148
Lassa Z148	Arenaviridae	II	10^−3^ ng	Lassa virus strain Z148
Lassa Acar	Arenaviridae	II	10^−1^ ng	No detection
Lassa Weller	Arenaviridae	II	10^−1^ ng	Lassa virus strain Weller
Lassa Pinneo	Arenaviridae	II	10^−1^ ng	Lassa virus strain Pinneo or Acar
Machupo Carvallo	Arenaviridae	III	10^−1^ ng	Machupo virus strain Carvallo
Machupo Chicava	Arenaviridae	III	10^−1^ ng	Machupo virus strain Chicava
Guanarito INH95551	Arenaviridae	III	10^−1^ ng	Guanarito virus strain INH-95551
Junin Rumero	Arenaviridae	III	10^−1^ ng	Junin virus strain Rumero
CCHFV^1^ 10200	Bunyaviridae	III	10^−1^ ng	CCHFV strain IbAr10200
Rift Valley fever	Bunyaviridae	II	10^−1^ ng	Rift Valley fever virus
Sandfly Sicilian	Bunyaviridae	IV	10^−1^ ng	No detection
Sandfly Naples	Bunyaviridae	IV	10^−1^ ng	Sandfly Naples strain NAMRU 840055
Toscana	Bunyaviridae	IV	10^−1^ ng	Toscana virus
Punta Toro	Bunyaviridae	IV	10^−1^ ng	No detection
Seoul	Bunyaviridae	IV	10^−1^ ng	Seoul virus
Hantaan	Bunyaviridae	IV	10^−1^ ng	No detection
Puumala	Bunyaviridae	N/A	10^−1^ ng	No detection
Sin nombre	Bunyaviridae	III	10^−1^ ng	Pulmonary syndrome hantavirus strain Convict Creek 107

1 CCHFV = Crimean-Congo hemorrhagic fever virus.

### Nucleic acid extraction

For bacteria and viruses, which were rated for handling in BSL-2 environment or higher rated, inactivated organisms, genomic DNA was extracted in NRL using the MasterPure DNA purification kit (Epicentre Technologies, Madison, WI) according to manufacturer's recommendations.

For some agents, which required BSL-3 facilities, bacterial genomic DNA was kindly provided by Dr. Ted Hadfield from Air Force Institute of Pathology (AFIP), Washington, DC.

Viral RNA was extracted using TRIzol LS (Invitrogen, Carlsbad, CA) at USAMRIID according to manufacturer's recommended protocol. The final pellet of product RNA was resuspended using 100 µl of RNase free water (Ambion, Austin, TX) and incubated at 65°C for 5 min.

### Quantification of nucleic acids

Bacterial genomic DNA preparations were quantified using NanoDrop ND1000 (Thermo Scientific Inc., Waltham, MA) spectrophotometer and genome copy number was calculated using the genome size and the DNA concentration. Viral DNA and RNA preparations, which also contained nucleic acids from cell culture, were subjected to quantitative real-time reverse transcription (RT)-PCR/PCR against concentration standards of the virus to determine the copy number of the viral templates. In some cases where standards were unavailable, the concentration of the virus was expressed in plaque forming units (pfu). For synthetic DNA templates, the DNA concentration was used to calculate the number of copies of the template based on the size of the DNA fragments.

### Chip processing and automatic sequence based identification

Microarray hybridization and processing, image scanning and processing were performed as previously described [18]. GeneChip Analysis Software v. 4.0 (Affymetrix, Santa Clara, CA) was used to produce FASTA output files. Final pathogen identification was performed using Computer-Implemented Biological Sequence Identifier (CIBSI) Version 2.0 software [28], an automatic pathogen identification algorithm based on nucleic acid sequence alignment, which was developed and tested in detail in previous studies [18], [19]. The NCBI BLAST and taxonomy databases used for CIBSI analysis were downloaded in October 2008. Due to the fact that sequence databases used by CIBSI are redundant and the nature of the available taxonomy database, the automated identifications made by this software were usually limited to the species level unless only a single sequence was the best scoring match. To achieve strain level discrimination when multiple sequences had the same best scoring match, the results were reviewed to determine if these sequences were in fact redundant and represented the same strain.

## Results

### Amplification-Primer Cocktail Optimization

**Figure 2:**
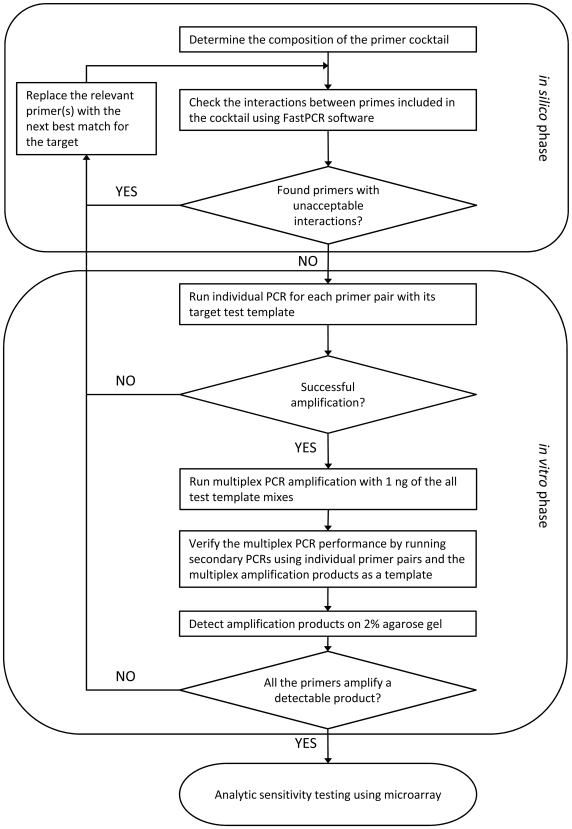
Procedure of optimization of primer cocktails. The diagram illustrates the procedure used to optimize the compositions of primer cocktails used for target amplification. The procedure consists of two stages. First stage was conducted using software for analysis of primer interactions and the second stage was carried out *in vitro* by testing of the performance of cocktails in multiplex PCR reactions.

As shown in Figure 2, the first step of the validation process was optimization of primers and primer mixes for specific gene targets on the chip. First, it was determined which targets will be amplified together, thus dictating which primer pairs end up in the same multiplex PCR mixture based on the criteria described in the methods section. In the next steps, a software script based on a selection algorithm developed by our group [29] was used to select primers from defined primer regions of each target based on criteria defined in previous studies [18]. A linker sequence was added to each primer in a cocktail and all of them were checked against each other for potential primer dimer interactions with FastPCR Professional v.5.2.71 (Primer Digital Ltd., http://www.biocenter.helsinki.fi/bi/Programs/fastpcr.htm, Helsinki, Finland). These processes were repeated until elimination of all primers having stretches of 8 bp or more matching with other primers in the same cocktail.

The second phase of primer cocktail optimization was run *in vitro* (Figure 2). All the primer pairs were tested individually with their corresponding targets. Any primer pairs that failed to amplify the targets were replaced. Next, the efficiency of complete cocktails was tested. In order to simulate the conditions that may occur in real samples, testing was conducted, in most cases, using mixtures of two to five different templates per reaction. Template composition of test samples for each PCR group is summarized in supporting Table S4. To avoid unnecessary cost, the initial testing of cocktails was conducted without using microarrays. The test samples with templates in concentration of 1 ng each per sample were amplified using multiplex PCR with the appropriate primer cocktails. The resulting products were purified and subjected to second stage individual specific PCR for each template present in the sample and analyzed on agarose gel.

Previous experience has indicated that if primers for a particular target are efficient enough to amplify specific products in quantity that is detectable on a gel then in most cases detection on microarray with LOD of at least 10^4^ target copies per test should be achieved. In the case of a negative result, the primers for that particular target were replaced and the modified cocktail was retested.

### Analytic sensitivity validation

Since only a few of the targeted biothreat agents can be obtained and processed in a BSL2 laboratory, efforts using natural genomic templates to demonstrate LOD for a majority of the targets on RPM-TEI chip were constrained. To validate the RPM-TEI assay's full capabilities for biothreat agent detection, artificially generated gene fragments (546–1200 bp) were used as an alternate means to estimate platform LOD (supporting Table S3).

The final sensitivity testing was conducted in two stages. First, samples containing mixed templates at 10^4^ copies per assay were prepared (for mix compositions see supporting Table S4) and run on microarrays. Templates for which detection at 10^4^ was not achieved were retested at 10^6^ copies per sample. The results of the sensitivity testing are summarized in Table 3 and the detailed results for each target are listed in supporting Table S2. It was found that 129 out of 187 test target templates (69%) were detected at 10^4^ copies, and 47 (25%) were detected at 10^6^ copies, while only 11 targets (6%) were found to have a LOD higher than 10^6^ template copies. Since most pathogens have two or more gene targets represented on the microarray and detection of any single target for a particular pathogen is all that is required for its successful detection, approximately 80% (66 out of 84) of the pathogens can be detected at 10^4^ genome copies. Remaining pathogens, with the single exception of *Cryptosporidium parvum* were found to achieve a limit of detection of 10^6^ copies.

**Table 3:** Summarized results of RPM-TEI sensitivity testing.

PCR group	LOD^1^ at 10^4^	LOD at 10^6^	LOD>10^6^	Total
I	28 (15)	16 (5)	5 (0)	49 (20)
II	32 (12)	12 (2)	0 (0)	44 (14)
III	42 (18)	1 (0)	1 (0)	44 (18)
IV	27 (21)	18 (10)	5 (1)	50 (32)
Total	129 (66)	47 (17)	11 (1)	187 (84)

1 LOD = limit of detection. The results refer to the number of *targets* with particular LOD. The results in parentheses refer to number of *pathogens* detected with particular LOD.

### Testing with whole genome templates of selected viruses

The performance of RPM-TEI was tested using a number of whole genome preparations of viruses to compare with validation results using synthetic templates. Due to “select agent” status of pathogens from which these genomic nucleic acid preparations were obtained, the experiments were conducted in the Virology Division of USAMRIID (Ft. Detrick, Frederick, MD). For the list of agents and detailed results see Table 2.

Initially four distinct viruses (Ebola Zaire, Ebola Reston, Lassa Josiah and Lassa Z148) each of them in four 10-fold dilutions (from 1 ng to 10^−3^ ng per microarray) were used to test the specificity and sensitivity of the RPM-TEI. It was estimated that 1 ng genomic RNA corresponds to approximately 10^5^ pfu, based on titration in cell culture. The microarray consistently provided correct detection and identification of these viruses, except for Lassa Josiah at the lowest tested concentration, which was only slightly below the detection threshold. Subsequently, further 10-fold serial dilutions of Ebola Zaire virus, to 10^−5^ ng, were used to assess the practical LOD. The results showed that LOD for genomic RNA preparation of this virus was between 10 and 1 pfu. These results indicate that establishing LOD with synthetic template is a valid alternative if genomic materials cannot be obtained.

In addition, several agents belonging to PCR group II through IV (most of the PCR group I targets were previously tested using genomic nucleic acid templates at NRL) were also tested. The testing was conducted using total genomic nucleic acids of 22 different viral agents in addition to the four initially tested. The viral template preparations used were at 0.1 ng per sample.

The RPM-TEI microarray was able to successfully detect the majority of viruses across all three tested groups. Most of the positively identified samples were correctly identified to the strain level. In addition, the microarrays were able to discriminate between closely related viral strains in a number of cases. RPM-TEI was able to distinguish between Zaire 1995 and Zaire Mayinga strains of Ebola virus, and correctly differentiated between Machupo virus strains Carvallo and Chicava. When testing several strains of Lassa viruses, correct unambiguous identifications were made for three distinct strains, Josiah, Z148 and Weller. In the case of Lassa virus Pinneo strain, the RPM-TEI identification narrowed it down to being one of two strains, Pinneo and Acar.

Of 26 agents tested in this series of experiments, 6 returned negative results. One of the negative samples was expected, since Puumala virus (a species of Hantavirus) was not represented on the chip. In the remaining five cases (Marburg Musoke, Lassa Acar, two Sandfly fever viruses: Sicilian and Punta Toro as well as Hantaan virus), the quality of the RNA preparation was considered to be the most likely explanation for the lack of detected agent sequence(s). At the time these experiments were conducted no other preparations of these agents were available.

## Discussion

This study demonstrates that RPM-TEI platform is able to achieve highly specific and sensitive detection of multiple biothreat agents in a single test. In contrast to contemporary methods used for microbial diagnostics and surveillance, RPM technology supports simultaneous detection and differential identification of hundreds of targets in a single diagnostic run. In addition, the resulting sequence information can be used to assess pathogenicity, mutations, virulence markers, and to differentiate detected agents from closely related species. This detailed information on the detected infectious agent may be invaluable for recognizing the false alarms caused by harmless confounders, and adequate risk management/exposure response planning.

Selection of diagnostic marker gene sequences as RPM detectors to be tiled on the microarray is critical to assay sensitivity and specificity. In the case of bacterial pathogens, it is relatively straightforward to find targets that cover all variants of the species. However, it is more difficult to ensure discrimination from near neighbor species so multiple targets are usually required (supporting Table S1).

For viruses, especially RNA viruses, the highly variable nature of their genomes warranted a multistage design process to select a minimal number of sequences for the detection and differential identification of known strains developed previously [20], [29]. The same strategy with some further modification was used when designing RPM-TEI. Testing of the RPM-TEI microarray conducted with several strains of different viral agents confirmed the general validity of this approach. The sequence information obtained in the testing process enabled very precise strain level identifications in many cases. We were able to discriminate between two Ebola Zaire strains (Zaire 1995 and Zaire Mayinga), whose genomes differ only by 2% on the nucleotide level and 0.6% on the protein level. Similar strain discrimination was obtained in case of Machupo virus strains Carvallo and Chicava [30] (sequence identity at 97% for segment S and 96% for segment L). Lassa virus is another excellent example of this capability. Out of 5 tested Lassa strains 3 were unambiguously identified to the strain level. In the case of Lassa Pinneo, strain identification could only be narrowed down to two possible strains Acar and Pinneo because their S genome segments (3.5 kb total length) differ by only 3 nucleotide changes, and these nucleotides are not represented by the probes used on the RPM-TEI array.

One of the most noteworthy and innovative parts of this work is the approach to the sensitivity testing of the microarray. Due to restricted access to the “select agents” that the microarray was designed to detect, a library of 142, plasmid-embedded synthetic target DNA fragments was used to conduct the analytical testing for most of the viral agents. This method of testing enabled us to carry out all of the validation experiments in a BSL2 laboratory. These sensitivity validation experiments differed from real world testing situations in a number of ways: they used DNA instead of RNA (majority of viral agents detected by RPM-TEI are RNA viruses) and the test templates contained isolated target sequences outside of the whole genome context. However, the aforementioned confirmatory experiments with full-length viral RNA genomic preparations have shown that this novel strategy is a suitable alternative for sensitivity validation purposes. Our previous experience with respiratory organisms has indicated that a LOD ∼10^4^ copies for the sensitivity validations provides the required detection sensitivity in real world clinical samples ∼10 pfu [19]. The results of this work indicate that LOD at ∼10^4^ copies using synthetic templates correlates with 10 pfu or less of the full length viral genomic preparations which we believe is the required target LOD of sensitivity validations. It remains for a complete study of clinical and environmental samples using the RPM-TEI and integration of clinical and epidemiological data before the performance will be fully established.

While the RPM chips demonstrate an excellent detection sensitivity and specificity for the majority of the targets, a few pathogens and toxin gene targets were detected with lower sensitivities. This was most likely caused by inefficient amplification at the multiplex PCR stage of detection. The primer selection process and amplification procedures for RPM-TEI are designed to minimize the impact of primer integrity, primer stability, and sample stability on detection capability but these can never be completely alleviated. Due to constraints on primer design resulting from the high level of multiplexing, it is unavoidable that there will be variable levels of amplification for different targets. A greater level of variability can be tolerated due to the RPM detection process but when it is too great it may lower sensitivity. In addition, mutations of target sequences are always a possibility that may reduce the efficiency of primer-binding sites resulting in inefficient amplification and detection failure. Furthermore, like all other molecular detection methods, the sensitivity of this assay is also dependent on the quality of front-end sample processing. Problems with sample preparation and/or storage may have contributed to the lack of identification of the 6 viral RNA preparations tested at 0.1 ng. The detection failure in a few of those cases was most likely the result of insufficient sample quality.

There are also limitations specific to the RPM technology that have been extensively discussed previously [18]. Chiefly, the limited space available on the microarrays requires making tradeoffs between breadth and depth of target coverage. This problem may be alleviated in future with availability of microarrays with greater densities.

Finally, it should be noted that although the list of targets included on the RPM-TEI chip was selected to maximize detection of agents important from a biodefense perspective, many of these pathogens are also known to be endemic in certain regions such as Central Africa for hemorrhagic fevers caused by filoviruses [31] or South America for hemorrhagic fevers caused by arenaviruses [32] and Dengue viruses [33]. For this reason the RPM-TEI assay may prove useful for diagnostics and epidemiologic investigations in the regions of the world affected by these agents. The sequence information generated from the RPM in conjunction with previously developed sequence analysis algorithm CIBSI can be easily interpreted to make serotype or strain identifications. This feature, the platform's high resolution, high throughput, and relatively modest cost per single detected pathogen provide support for use of the RPM-TEI as a diagnostic and surveillance tool in regional reference laboratories. Efforts continue to test the utility of this assay using samples having more diverse biological origins and pathogen content.

## Supporting Information

Table S1 List of detector sequences used to design RPM-TEI. Includes their origin and amplification related information.
(0.04 MB XLS)

Table S2 Detailed results of RPM-TEI sensitivity testing.
(0.03 MB XLS)

Table S3 Complete list of synthetic templates used for RPM-TEI validation.
(0.03 MB XLS)

Table S4 Composition of template mixes used for sensitivity testing of RPM-TEI microarray.
(0.02 MB XLS)

Figure S1 Relative allocation of space on the RPM-TEI for detected pathogens
(0.59 MB PPT)

Text S1 Detector sequence selection for highly variable viruses using Lassa as an example.
(0.08 MB DOC)

## References

[1] LaForce FM (1994). Anthrax.. Clin Infect Dis.

[2] Das R, Hammamieh R, Neill R, Ludwig GV, Eker S (2008). Early indicators of exposure to biological threat agents using host gene profiles in peripheral blood mononuclear cells.. BMC Infect Dis.

[3] Amann RI, Ludwig W, Schleifer KH (1995). Phylogenetic identification and in situ detection of individual microbial cells without cultivation.. Microbiol Rev.

[4] Rotz LD, Khan AS, Lillibridge SR, Ostroff SM, Hughes JM (2002). Public health assessment of potential biological terrorism agents.. Emerg Infect Dis.

[5] Radnedge L, Gamez-Chin S, McCready PM, Worsham PL, Andersen GL (2001). Identification of nucleotide sequences for the specific and rapid detection of Yersinia pestis.. Appl Environ Microbiol.

[6] Kim K, Seo J, Wheeler K, Park C, Kim D (2005). Rapid genotypic detection of Bacillus anthracis and the Bacillus cereus group by multiplex real-time PCR melting curve analysis.. FEMS Immunol Med Microbiol.

[7] Wilson WJ, Erler AM, Nasarabadi SL, Skowronski EW, Imbro PM (2005). A multiplexed PCR-coupled liquid bead array for the simultaneous detection of four biothreat agents.. Mol Cell Probes.

[8] Pingle MR, Granger K, Feinberg P, Shatsky R, Sterling B (2007). Multiplexed identification of blood-borne bacterial pathogens by use of a novel 16S rRNA gene PCR-ligase detection reaction-capillary electrophoresis assay.. J Clin Microbiol.

[9] Liu RH, Munro SB, Nguyen T, Siuda T, Suciu D (2006). Integrated Microfluidic CustomArray Device for Bacterial Genotyping and Identification.. JALA.

[10] Dunbar SA, Jacobson JW (2007). Parallel processing in microbiology: Detection of infectious pathogens by Luminex xMAP multiplexed suspension array technology.. Clinical Microbiology Newsletter.

[11] Dunbar SA, Jacobson JW (2007). Quantitative, multiplexed detection of Salmonella and other pathogens by Luminex xMAP suspension array.. Methods Mol Biol.

[12] Briese T, Palacios G, Kokoris M, Jabado O, Liu Z (2005). Diagnostic system for rapid and sensitive differential detection of pathogens.. Emerg Infect Dis.

[13] Blais DR, Alvarez-Puebla RA, Bravo-Vasquez JP, Fenniri H, Pezacki JP (2008). Multiplex pathogen detection based on spatially addressable microarrays of barcoded resins.. Biotechnol J.

[14] Nakamura S, Maeda N, Miron IM, Yoh M, Izutsu K (2008). Metagenomic diagnosis of bacterial infections.. Emerg Infect Dis.

[15] Nakamura S, Yang C-S, Sakon N, Ueda M, Tougan T (2009). Direct Metagenomic Detection of Viral Pathogens in Nasal and Fecal Specimens Using an Unbiased High-Throughput Sequencing Approach.. PLoS ONE.

[16] Berthet N, Dickinson P, Filliol I, Reinhardt AK, Batejat C (2007). Massively parallel pathogen identification using high-density microarrays.. Microbial Biotechnology.

[17] Lin B, Wang Z, Vora GJ, Thornton JA, Schnur JM (2006). Broad-spectrum respiratory tract pathogen identification using resequencing DNA microarrays.. Genome Res.

[18] Lin B, Blaney KM, Malanoski AP, Ligler AG, Schnur JM (2007). Using a resequencing microarray as a multiple respiratory pathogen detection assay.. J Clin Microbiol.

[19] Lin B, Malanoski AP, Wang Z, Blaney KM, Ligler AG (2007). Application of broad-spectrum, sequence-based pathogen identification in an urban population.. PLoS ONE.

[20] Wang Z, Malanoski AP, Lin B, Kidd C, Long NC (2008). Resequencing microarray probe design for typing genetically diverse viruses: human rhinoviruses and enteroviruses.. BMC genomics.

[21] Gingeras TR, Ghandour G, Wang E, Berno A, Small PM (1998). Simultaneous genotyping and species identification using hybridization pattern recognition analysis of generic Mycobacterium DNA arrays.. Genome Res.

[22] Taitt CR, Malanoski AP, Lin B, Stenger DA, Ligler FS (2008). Discrimination between biothreat agents and ‘near neighbor’ species using a resequencing array.. FEMS Immunol Med Microbiol.

[23] Boonham N, Fisher T, Mumford RA (2005). Investigating the specificity of real-time PCR assays using synthetic oligonucleotides.. J Virol Methods.

[24] Christensen TM, Jama M, Ponek V, Lyon E, Wilson JA (2007). Design, development, validation, and use of synthetic nucleic acid controls for diagnostic purposes and application to cystic fibrosis testing.. J Mol Diagn.

[25] Charrel RN, La Scola B, Raoult D (2004). Multi-pathogens sequence containing plasmids as positive controls for universal detection of potential agents of bioterrorism.. BMC Microbiol.

[26] Carrera M, Sagripanti JL (2009). Artificial plasmid engineered to simulate multiple biological threat agents.. Appl Microbiol Biotechnol.

[27] Malanoski AP, Lin B, Stenger DA (2008). A model of base-call resolution on broad-spectrum pathogen detection resequencing DNA microarrays.. Nucleic Acids Res.

[28] Malanoski AP, Lin B, Wang Z, Schnur JM, Stenger DA (2006). Automated identification of multiple micro-organisms from resequencing DNA microarrays.. Nucleic Acids Res.

[29] Lin B, Malanoski AP, Wang Z, Blaney KM, Long NC (2009). Universal detection and Identification of avian Influenza using resequencing microarrays.. J Clin Microbiol.

[30] Cajimat MN, Milazzo ML, Rollin PE, Nichol ST, Bowen MD (2009). Genetic diversity among Bolivian arenaviruses.. Virus Res.

[31] Gonzalez JP, Pourrut X, Leroy E (2007). Ebolavirus and other filoviruses.. Curr Top Microbiol Immunol.

[32] Tesh RB (2002). Viral hemorrhagic fevers of South America.. Biomedica.

[33] Teixeira MG, Costa MC, Coelho G, Barreto ML (2008). Recent shift in age pattern of dengue hemorrhagic fever, Brazil.. Emerg Infect Dis.

