# Supplementary material for: Testing and Validation of High Density Resequencing Microarray for Broad Range Biothreat Agents Detection
Source: PLoS One. 2009 Aug 11;4(8):e6569. doi: 10.1371/journal.pone.0006569 (PMC2719057; doi:10.1371/journal.pone.0006569)
Supplement: Figure S1 — Relative allocation of space on the RPM-TEI for detected pathogens (0.59 MB PPT) [file pone.0006569.s005.ppt]

## Slide 1
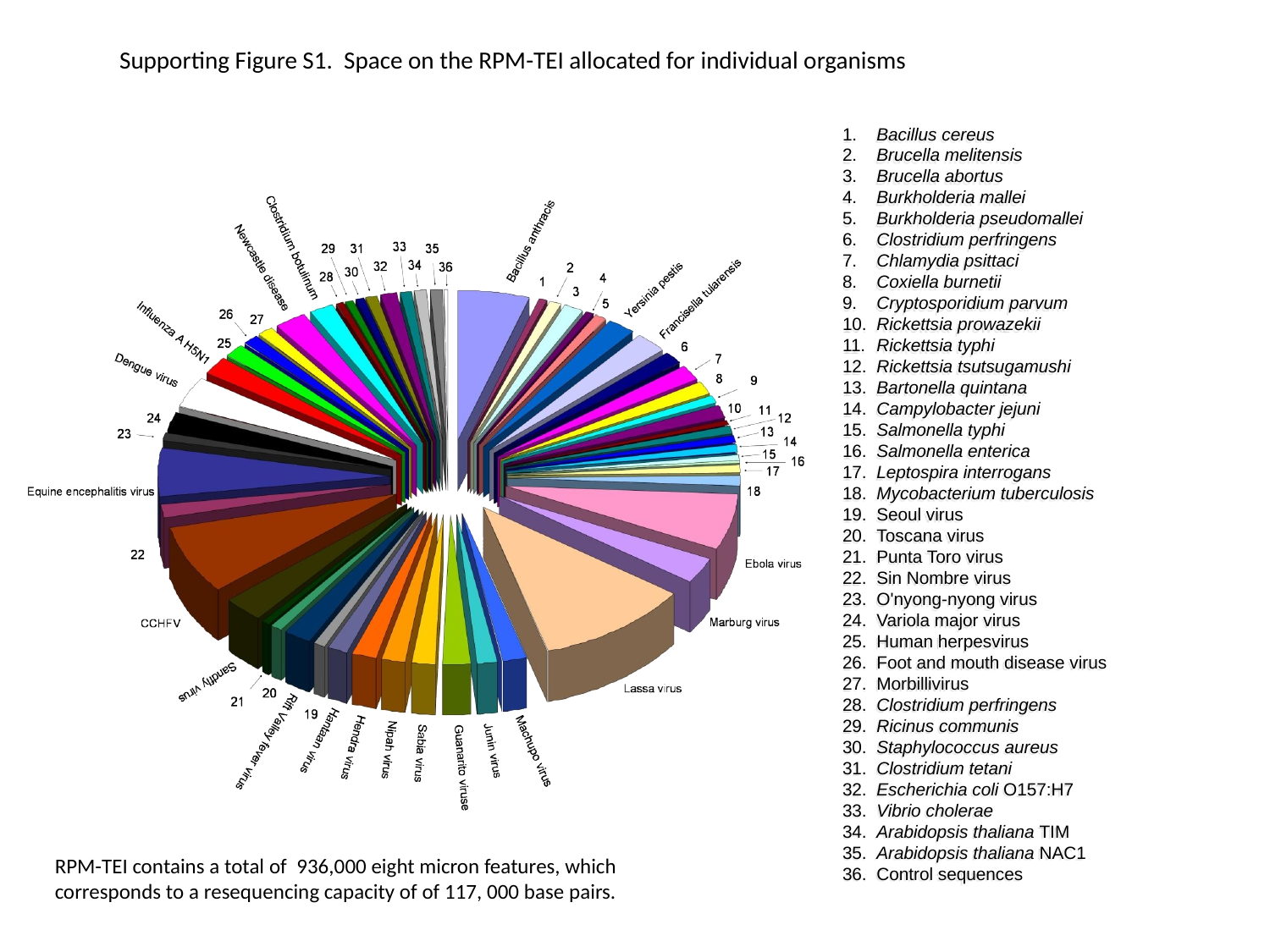

Supporting Figure S1. Space on the RPM-TEI allocated for individual organisms
1.	Bacillus cereus
2.	Brucella melitensis
3. 	Brucella abortus
4. 	Burkholderia mallei
5.	Burkholderia pseudomallei
6.	Clostridium perfringens
7.	Chlamydia psittaci
8.	Coxiella burnetii
9.	Cryptosporidium parvum
10.	Rickettsia prowazekii
11.	Rickettsia typhi
12.	Rickettsia tsutsugamushi
13.	Bartonella quintana
14.	Campylobacter jejuni
15.	Salmonella typhi
16.	Salmonella enterica
17.	Leptospira interrogans
18.	Mycobacterium tuberculosis
19.	Seoul virus
20.	Toscana virus
21.	Punta Toro virus
22.	Sin Nombre virus
23.	O'nyong-nyong virus
24.	Variola major virus
25.	Human herpesvirus
26.	Foot and mouth disease virus
27.	Morbillivirus
28.	Clostridium perfringens
29.	Ricinus communis
30.	Staphylococcus aureus
31.	Clostridium tetani
32.	Escherichia coli O157:H7
33.	Vibrio cholerae
34.	Arabidopsis thaliana TIM
35.	Arabidopsis thaliana NAC1
36.	Control sequences
RPM-TEI contains a total of 936,000 eight micron features, which corresponds to a resequencing capacity of of 117, 000 base pairs.
