# Supplementary material for: Testing and Validation of High Density Resequencing Microarray for Broad Range Biothreat Agents Detection
Source: PLoS One. 2009 Aug 11;4(8):e6569. doi: 10.1371/journal.pone.0006569 (PMC2719057; doi:10.1371/journal.pone.0006569)
Supplement: Text S1 — Detector sequence selection for highly variable viruses using Lassa as an example. (0.08 MB DOC) [file pone.0006569.s006.doc]

**Design of diagnostic sequences for detection of Lassa viruses.**

Lassa viruses are noted for high (24%) genomic nucleotide sequence diversity [1]. Therefore we selected this group as an example to illustrate improvements to the RPM microarray design process. There are six steps in the selection of diagnostic target sequences in this modified procedure

In the first step, nucleotide sequences related to all Lassa viruses were downloaded from GenBank (137 sequences at the time of design March 2007) and used as a Lassa virus specific database (LV_db). In the second step, three genes: LP and GP from encoded in the L segment, and NP, encoded in the S segment, were selected as targets to include each segment of the Lassa genome and as genes having different levels of interspecific variability. These steps are identical to the process that was originally developed.

The third step represents the first change in the procedure. A sequence with size between 0.5 and 2 kb for each gene was selected from the LV_db and the blastn algorithm was used to find all matching sequences present in the LV-db with E-value < 1e-20. Qualifying sequences were retrieved from the LV_db with a perl script, trimmed to the equivalent lengths (around 700 bp) and aligned using the ClustalW algorithm. A neighbor-joining phylogenetic tree was then generated from these sequences. In the fourth step, sequences from each tree branch were compared with those from neighboring branches. These pair-wise comparisons generated several distinct clusters for which each sequence in a particular cluster had > 90% nucleotide identity with every other sequence in that cluster. For example, the GP sequences were finally classified as five clusters based on sequence homologies as shown in the diagram, below.


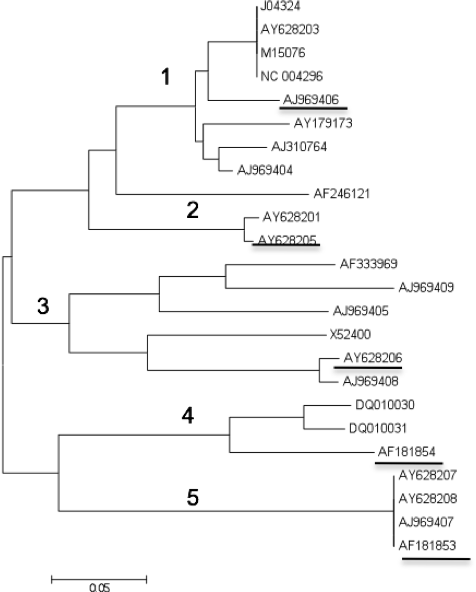


**Figure M1-1.** Classification of GP sequences from Lassa viruses. Cluster 1-5 were identified based on sequence homology and sequence within each cluster have 90% identity. Sequences chosen as probes were underlined.

A single diagnostic sequence was then selected to represent each cluster. The final steps return to the previously described process, where the selected diagnostic sequences are validated to assure that all known variants in each cluster can be detected [2,3]. As part of this step, if the selected sequence was not found to provide complete coverage of a cluster, additional sequences were added until detection of all sequences in the cluster was assured. As a result of this modified procedure, we selected 3 LP sequences, 5 GP sequences and 11 NP sequences to achieve complete coverage of all known Lassa strains.

**References:**

1. Bowen MD, Rollin PE, Ksiazek TG, Hustad HL, Bausch DG, et al. (2000) Genetic diversity among Lassa virus strains. J Virol 74: 6992-7004.

2. Malanoski AP, Lin B, Stenger DA (2008) A model of base-call resolution on broad-spectrum pathogen detection resequencing DNA microarrays. Nucleic Acids Res 36: 3194-3201.

3. Wang Z, Malanoski AP, Lin B, Kidd C, Long NC, et al. (2008) Resequencing microarray probe design for typing genetically diverse viruses: human rhinoviruses and enteroviruses. BMC genomics 9: 577.
